# Association between Gray and White Matter Lesions and Its Involvement in Clinical Symptoms of Alzheimer’s-Type Dementia

**DOI:** 10.3390/jcm12247642

**Published:** 2023-12-12

**Authors:** Taizen Nakase, Benjamin Thyreau, Yasuko Tatewaki, Naoki Tomita, Yumi Takano, Michiho Muranaka, Yasuyuki Taki

**Affiliations:** 1 Department of Aging Research & Geriatric Medicine, Institute of Development, Aging and Cancer, Tohoku University, Sendai 980-8575, Japan; yasuko.tatewaki.a7@tohoku.ac.jp (Y.T.); naoki.tomita.b1@tohoku.ac.jp (N.T.); yasuyuki.taki.c7@tohoku.ac.jp (Y.T.); 2 Smart Aging Research Center, Tohoku University, Sendai 980-8575, Japan; benjamin.thyreau.a5@tohoku.ac.jp

**Keywords:** dementia, retrospective study, cortical lesion, medullary lesion, cognitive impairment, neuropsychological symptom

## Abstract

Background: Not only gray matter lesions (GMLs) but also white matter lesions (WMLs) can play important roles in the pathology of Alzheimer’s disease (AD). The progression of cognitive impairment (CI) and behavioral and psychological symptoms of dementia (BPSD) might be caused by a concerted effect of both GML and WML. Objective: This study aimed to investigate the association between GML and WML and how they are involved in the symptoms of CI and BPSD in dementia patients by means of imaging technology. Methods: Patients in our memory clinic, who were diagnosed with AD-type dementia or amnestic mild cognitive impairment (aMCI) and had undergone both single-photon emission computed tomography (SPECT) and brain MRI, were consecutively enrolled (*n* = 156; 61 males and 95 females; 79.8 ± 7.4 years old). Symptoms of CI and BPSD were obtained from patients’ medical records. For the analysis of GMLs and WMLs, SPECT data and MRI T1-weighted images were used, respectively. This study followed the Declaration of Helsinki, and all procedures were approved by the institutional ethics committee. Results: According to a multivariate analysis, disorientation and disturbed attention demonstrated a relationship between the precuneus and WMLs in both hemispheres. Hyperactivity in BPSD showed multiple correlations between GMLs on both sides of the frontal cortex and WMLs. Patients with aMCI presented more multiple correlations between GMLs and WMLs compared with those with AD-type dementia regarding dementia symptoms including BPSD. Conclusion: The interaction between GMLs and WMLs may vary depending on the symptoms of CI and BPSD. Hyperactivity in BPSD may be affected by the functional relationship between GMLs and WMLs in the left and right hemispheres. The correlation between GMLs and WMLs may be changing in AD-type dementia and aMCI.

## 1. Introduction

In the human brain, the default mode network (DMN) has been reported to play an important role in maintaining cognitive function [1]. The anterior cingulate cortex, medial prefrontal cortex, posterior cingulate cortex, and inferior parietal cortex are included in the DMN [2]. Moreover, a disconnection of the DMN, especially in the posterior cingulate cortex, has been observed in Alzheimer’s disease (AD) patients [3]. Connectivity between different cortical areas in the DMN can be affected by cerebral white matter lesions (WMLs), meaning that WMLs may play an important role in the pathogenesis of AD [4]. Pathologically, WMLs may be observed in the very early stages of AD [5]. In fact, the expansion of WMLs before a diagnosis of AD has been reported in the analysis of hereditary AD patients [6]. Regardless of the amount, WMLs are frequently observed in brain images obtained from elderly persons [7]. In terms of the etiology of WMLs, several pathological conditions can be involved, i.e., the damage of endothelial cells in brain arterioles as small vessel disease, dysfunction of the blood–brain barrier caused by chronic hypoperfusion or the inflammatory response, demyelination, and loss of neuronal axons [8]. Since WMLs frequently exist in AD pathology, it is difficult to identify the actual cause of WMLs without pathological analysis. Nevertheless, the decline in cognitive function in AD was accelerated by WMLs [9,10]. The increase in WMLs was enhanced by constipation, and this observation was accompanied by a worsening of cognitive impairment in AD patients [11]. 

In the pathology of AD brain, hypofunction of gray matter (GM) has been reported to precede cortical atrophy [12]. Amyloid deposition is mainly observed in GM [13]. From the viewpoint of dementia symptoms, both gray matter lesions (GMLs) and WMLs might be involved in the pathogenesis of cognitive impairment and behavioral and psychological symptoms of dementia (BPSD) in dementia patients [14]. It is worthwhile to simultaneously assess GMLs and WMLs to understand the origin of BPSD as well as cognitive symptoms. In this study, we evaluated GMLs as the hypoperfusion of regional GM using ^123^I-iodoamphetamine–single-photon emission tomography (IMP-SPECT) and investigated its correlation with WMLs regarding dementia symptoms in patients with AD-type dementia and amnestic mild cognitive impairment (aMCI).

## 2. Methods

All procedures of this study were approved by the Independent Ethics Committee, the Tohoku University School of Medicine (#2021-1-385). The review board waived the need for patient consent, as this was a retrospective study, and all data were deidentified. This study was conducted in accordance with the principles of the Declaration of Helsinki.

We screened consecutive patients who were clinically diagnosed with AD-type dementia or aMCI by registered neurologists or gerontologists following the guidelines [11,15] in our memory clinic from April 2017 to March 2020 (*n* = 175). We used “AD-type dementia” for the diagnosis of enrolled patients because our diagnosis follows classic guidelines that do not include AD biomarkers, such as amyloid-β-positive cerebrospinal fluid or positron emission tomography. Those patients who had undergone both brain magnetic resonance imaging (MRI: Intera Achieva 3.0T Quasar Dual, Philips, Amsterdam, The Netherlands) and IMP-SPECT were enrolled in this study (*n* = 156; 61 males and 95 females; average (SD: standard deviation) age was 79.8 (7.4) years old). Personal educational background, past medical history, comorbidities (such as hypertension, hyperlipidemia, diabetes mellitus, and cardiac disease), and prescribed medicines were extracted from the medical records. Laboratory data referred to the blood examination at the first visit. Cognitive impairment was assessed using the Mini-Mental State Examination Japanese version (MMSE-J) performed within 1 month of the first visit. Symptoms of cognitive impairment were evaluated based on the subcategories of MMSE-J and were classified into five categories: (1) disorientation, referring to the disorientation of time and location (scored from 0 (none) to 10 (severe)); (2) disturbed attention, referring to the reduced capability of mental arithmetic (scored from 0 (none) to 5 (severe)); (3) memory disturbance, referring to disturbed delayed recall of three words (scored from 0 (none) to 3 (severe)); (4) disorder of spatial perception, referring to the disturbed writing of the double pentagon (scored as 0 (none) or 1 (positive)); and (5) impairment of language skill, referring to disturbed repeating of a sentence and writing skill (scored from 0 (none) to 2 (severe)). Assessment of BPSD was based on the subcategories of the Neuropsychiatric Inventory-Questionnaire (NPI-Q). The NPI-Q is a questionnaire-format test that is completed by the caregiver of the patient [16]. It can rate the severity of each symptom of BPSD and the burden of caregivers by scoring across 12 domains; that is, delusions, hallucinations, agitation, depression, anxiety, euphoria, apathy, disinhibition, irritability, motor disturbance, nighttime behaviors, and appetite. Then, we classified BPSD into five categories: (1) hypoactivity, accounting for apathy and depression; (2) hyperactivity, accounting for disinhibition, irritability, and repetitive activities; (3) hallucination/delusion, including visual and verbal hallucinations, and persecutory or jealousy delusion; (4) abnormal behavior, including agitation, anxiety, euphoria, and change in the type of food consumed; and (5) disturbed circadian rhythm, accounting for hypersomnia in the daytime and waking during the night. Brain MRI T1-weighted images (T1-WI) were adopted for the calculation of the volume of cerebral WMLs. WMLs were separately assessed in the periventricular region (PVWML) of the left/right anterior horn, the PVWML of the left/right posterior horn, the deep white matter region (DWML) in the left/right frontal lobe, and the DWML in the left/right parieto-occipital lobe. Calculations were performed by an in-house pipeline based on the Statistical Parametric Mapping (SPM)–Lesion Segmentation Tool toolbox (https://www.applied-statistics.de/lst.html, accessed on 13 February 2023). The cerebral areas of the 2D images were measured and summed for all areas to obtain the whole cerebral volume and presented in units of mL. Magnetic resonance angiography (MRA) was used to analyze the vessel stenosis in the major brain arteries. Alteration of regional cerebral blood flow (rCBF) assessed with IMP-SPECT was used for the analysis of GMLs. The severity of GMLs was evaluated with the Z-score of three-dimensional stereotactic surface projection (3D-SSP) analysis, which was calculated using the implemented software in the SPECT system (Symbia-E, Siemens Healthineers, Erlangen, Germany). The regions of interest of GMLs were the left/right superior frontal cortex, left/right middle frontal cortex, left/right medial frontal cortex, left/right medial temporal cortex, left/right posterior cingulate cortex, and left/right precuneus.

### Statistical Analysis

Data are presented as means ± standard deviation (SD) or as a number and percentage. Between AD-type dementia and aMCI, the prevalence of risk factors and severity of cognitive impairment were compared using Student’s *t*-test, and the percentage of BPSD was compared using Pearson’s *t*-test. The associations between the volume of WMLs and atherosclerotic risk factors were assessed via multivariate analysis. Logistic regression analysis was used for the assessment of the correlations between subcategories of cognitive impairment and GMLs or WMLs and for the assessment of the correlations between subcategories of BPSD and GMLs or WMLs. Pearson’s correlation coefficient is presented as the “r” value. Spearman’s rank correlation coefficient was used for the investigation of the relationship between GMLs and WMLs in each subcategory of cognitive impairment and in each subcategory of BPSD. Spearman’s rank correlation coefficient is presented as the “rs” value. JMP Pro15 software was used for the analysis. A *p*-value of less than 0.05 was defined as significant.

## 3. Results

### 3.1. Clinical Characteristics

As shown in Table 1, the percentage of patients with AD-type dementia was 65.4%. The frequencies of atherosclerotic risk factors, such as hypertension, hyperlipidemia, diabetes mellitus, and cardiac diseases, were 67.3%, 37.8%, 26.3%, and 10.9%, respectively. The mean age of aMCI patients was relatively younger than that of AD-type dementia patients. Comorbid diabetes was significantly more frequent in AD-type dementia than aMCI. The mean (SD) score of MMSE-J was 22.0 (4.1). The mean (SD) score of disorientation, disturbed attention, memory disturbance, disorder of spatial perception, and impairment of language skill were 2.2 (2.1), 2.3 (1.5), 2.1 (1.0), 0.2 (0.4), and 0.2 (0.4), respectively. Four out of five subcategories in cognitive impairment were significantly more severe in AD-type dementia than aMCI. Moreover, the frequency of BPSD was 56.4% in all patients. Among subcategories of BPSD, hypoactivity was the most common symptom (34.6%). Hypoactivity and hallucination/delusion were more frequent in AD-type dementia than aMCI.

### 3.2. Backgrounds of White Matter Lesions

**Figure 1:**
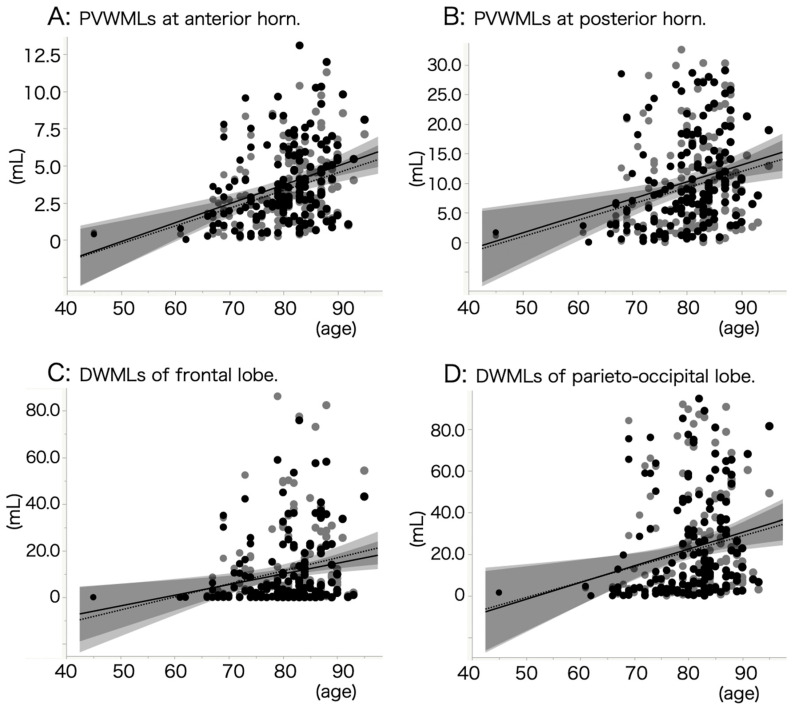
Correlation between severity of WMLs and age. There was a significant correlation between age and PVWML volume in anterior horn of lateral ventricle (**A**). The formula of regression line on the left is Y = 119.5X − 6240; *r* = 0.361; *p* < 0.001. The formula on the right is Y = 127.7X − 6516; *r* = 0.346; *p* < 0.001. There was a significant correlation between age and PVWML volume in posterior horn of lateral ventricle (**B**). The formula of regression line on the left is Y = 27.4X − 1272; *r* = 0.245; *p* = 0.002. The formula on the right is Y = 28.6X − 1268; *r* = 0.265; *p* = 0.001. There was a significant correlation between age and DWML volume in frontal lobe (**C**). The formula of regression line on the left is Y = 56.0X − 3345; *r* = 0.245; *p* = 0.003. The formula on the right is Y = 45.8X − 2658; *r* = 0.224; *p* = 0.004. There was a significant correlation between age and DWML volume in parieto-occipital lobe (**D**). The formula of regression line on the left is Y = 74.0X − 3787; *r* = 0.224; *p* = 0.006. The formula on the right is Y = 80.4X − 4193; *r* = 0.245; *p* = 0.003. Gray and black dots indicate data for left and right sides, respectively. Dotted and solid lines indicate the regression lines of left and right sides, respectively.

The severity of WMLs was assessed by comparing with age and MMSE-J score. There was a significant correlation between the severity of WMLs and age in all four regions (Figure 1A–D). However, no correlation was observed between the severity of WMLs and MMSE-J scores in all four regions (data not shown). Multivariate analysis was adopted to assess the involvement of atherosclerotic risk factors in the severity of WMLs (Table 2). There were significant correlations between PVWML in the left and right anterior horn and hypertension (*r* = 0.2444, *p* = 0.0021, and *r* = 0.2273, *p* = 0.0043, respectively). Those otsignificances were only observed in aMCI patients when patients with AD-type dementia and aMCI were separately analyzed. Moreover, left and right parieto-occipital DWMLs were significantly correlated with hypertension in aMCI patients.

### 3.3. Connectivity between Symptoms and Brain Lesions

Logistic regression analysis was adopted to evaluate the involvement of GMLs and WMLs in subcategories of cognitive impairment (Supplementary Table S1). Disorientation was negatively correlated with the Z-score in the right medial temporal cortex (*r* = −0.9594; *p* = 0.0010). Disturbed attention was positively correlated with the volume of the PVWML in the right posterior horn, positively correlated with the Z-score in the left superior frontal cortex, negatively in the left middle frontal cortex, and positively in the left posterior cingulate cortex (*r* = 0.0011, *p* = 0.038; *r* = 1.5469, *p* = 0.037; *r* = −1.4529, *p* = 0.0036; and *r* = 0.6934, *p* = 0.0456, respectively). Memory disturbance was negatively correlated with the Z-score in the left middle frontal cortex (*r* = −1.3585; *p* = 0.0232). Disorder of spatial perception was negatively correlated with the volume of PWMLs in the right anterior horn (*r* = −0.0007; *p* = 0.0329) and positively correlated with the Z-score in the right middle frontal cortex (*r* = 1.8756; *p* = 0.0490). No significant correlation was observed regarding the impairment of language skill.

Logistic regression analysis was also adopted to evaluate the involvement of GMLs and WMLs in subcategories of BPSD (Supplementary Table S2). Hallucination/delusion was positively correlated with the volume of the left frontal DWMLs and negatively to the volume of the right frontal DWMLs (*r* = 0.0014, *p* = 0.0298, and *r* = −0.0016, *p* = 0.0324, respectively). Abnormal behavior was positively correlated with the Z-score in the left medial frontal cortex (*r* = 5.6741; *p* = 0.0461). Hypoactivity and hyperactivity did not show any correlation with GMLs and WMLs in this analysis. Because the number of patients with disturbed circadian rhythms was only two, statistical analysis was not performed.

### 3.4. Correlations between Gray Matter Lesions and White Matter Lesions

**Figure 2:**
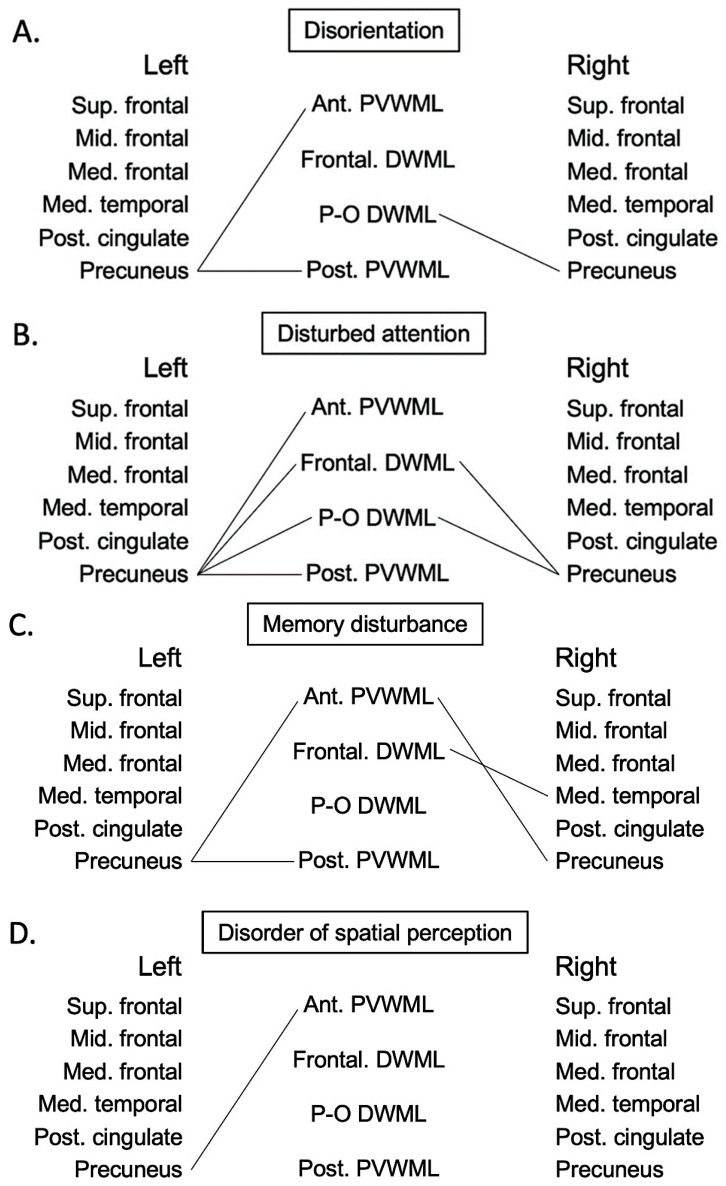

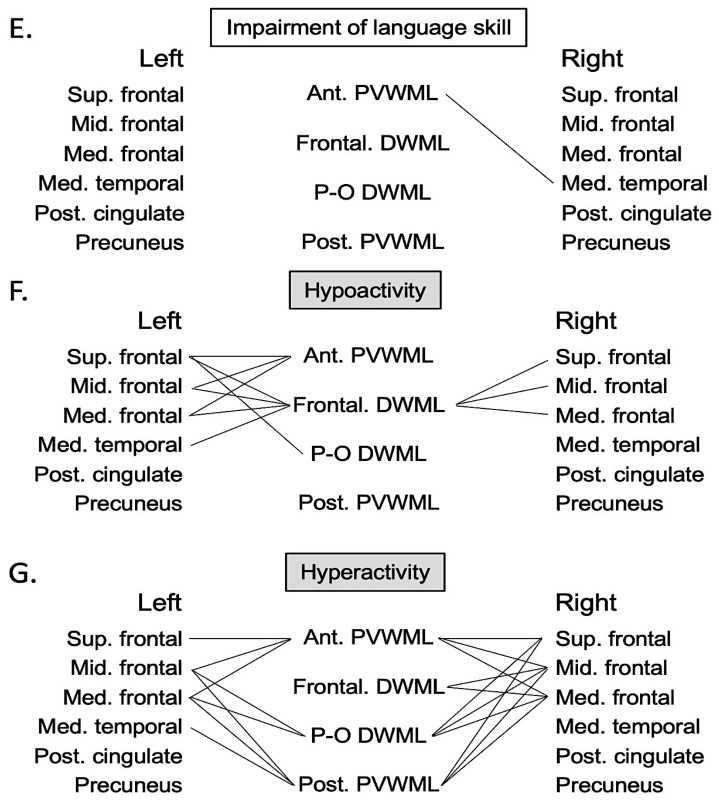
Significant correlations between GMLs and WMLs in each symptom (**A**) disorientation, (**B**) disturbed attention, (**C**) memory disturbance, (**D**) disorder of spatial perception, (**E**) impairment of language skill, (**F**) hypoactivity, and (**G**) hyperactivity. Significant correlation is shown by solid line between two lesions. Statistical data of each line are presented in Supplementary Tables. Sup.: superior; mid.: middle; med.: medial; post.: posterior; ant.: anterior; P-O: parieto-occipital.

Spearman’s rank correlation coefficient was adopted to evaluate the connectivity between GMLs and WMLs in each symptom of subcategories of cognitive impairment (Figure 2 and Supplementary Table S3). As for disorientation, there were significant correlations between the left precuneus and the PVWMLs in the left anterior horn (rs = −0.1967; *p* = 0.0360), between the left precuneus and the PVWMLs in the left posterior horn (rs = −0.2060; *p* = 0.0279), and between the right precuneus and the right parieto-occipital DWMLs (rs = −0.1993; 0.0336). As for disturbed attention, there were significant correlations between the left precuneus and all four of the WMLs (anterior horn PVWML rs = −0.2349, *p* = 0.0076; posterior horn PVWML rs = −0.2191, *p* = 0.0129; frontal DWML rs = −0.1912, *p* = 0.0306; and parieto-occipital DWML rs = −0.2199, *p* = 0.0126), between the right precuneus and the right frontal DWMLs (rs = −0.2221; *p* = 0.0117), and between the right precuneus and the right parieto-occipital DWMLs (rs = −0.2129; *p* = 0.0158). As for memory disturbance, significant correlations were observed between the left precuneus and the PVWMLs in the left anterior and posterior horn (rs = −0.1893, *p* = 0.0273, and rs = −0.1762, *p* = 0.0401, respectively), between the right precuneus and the PVWMLs in the right anterior horn (rs = −0.1725; *p* = 0.0446), and between the right medial temporal cortex and the frontal DWMLs (rs = 0.1756; *p* = 0.0409). As for disorder of spatial perception, a significant correlation was observed between the left precuneus and the PVWMLs in the left anterior horn (rs = −0.4231; *p* = 0.0279). As for impairment of language skill, a significant correlation was observed between the right medial temporal cortex and the PVWMLs in the right anterior horn (rs = −0.4165; *p* = 0.0429).

When patients with AD-type dementia and aMCI were separately analyzed (Supplementary Table S4), disorientation showed a significant correlation between the right precuneus and the right parieto-occipital DWMLs only in aMCI patients (rs = −0.4924; *p* = 0.0067). Disturbed attention showed significant multiple correlations between GMLs and WMLs in AD-type dementia and aMCI, the same as in the overall analysis. Memory disturbance a significant correlation between the right precuneus and the right parieto-occipital DWMLs only in aMCI patients (rs = −0.4036; *p* = 0.0073). As for disorder of spatial perception, patients with aMCI showed significant correlations between the left superior frontal cortex and the left parieto-occipital DWMLs (rs = −0.8857; *p* = 0.0188), between the left medial temporal cortex and the PVWMLs in the left posterior horn or the left parieto-occipital DWMLs (rs = −0.8117, *p* = 0.0499, and rs = −0.8986, *p* = 0.0149, respectively), and between the right precuneus and the right parieto-occipital DWMLs (rs = −0.8857; *p* = 0.0188). Because the number of patients with impairment of language skill was small, statistical data were not correctly obtained.

Spearman’s rank correlation coefficient was adopted to evaluate the connectivity between GMLs and WMLs in each symptom of subcategories of BPSD (Figure 2 and Supplementary Table S5). As for hypoactivity, there were significant correlations between the left superior frontal cortex and three of four WMLs (anterior horn PVWML rs = 0.3079, *p* = 0.0249; frontal DWML rs = 0.3684, *p* = 0.0066; and parieto-occipital DWML rs = 0.3141, *p* = 0.0220), between the left middle frontal cortex and the PVWMLs in the left anterior horn and the left frontal DWMLs (rs = 0.2976, *p* = 0.0304, and rs = 0.3553, *p* = 0.0090, respectively), between the left medial frontal cortex and the PVWMLs in the left anterior horn and the left frontal DWMLs (rs = 0.2820, *p* = 0.0408, and rs = 0.3368, *p* = 0.0137, respectively), between the left medial temporal cortex and the left frontal DWMLs (rs = 0.3023; *p* = 0.0278), between the right superior, middle, and medial frontal cortices and the right frontal DWMLs (rs = 0.3010, *p* = 0.0208; rs = 0.3065, *p* = 0.0256; and rs = 0.3181, *p* = 0.0203, respectively). As for hyperactivity, there were significant correlations between the left superior frontal cortex and the left PVWMLs in the anterior horn (rs = −0.4044; *p* = 0.0295), between the left middle frontal cortex and three of four left WMLs (anterior horn PVWML rs = −0.5197, *p* = 0.0039; posterior horn PVWML rs = −0.5158, *p* = 0.0042; and parieto-occipital DWML rs = −0.4039, *p* = 0.0298), between the left medial frontal cortex and three of four left WMLs (anterior horn PVWML rs = −0.5030, *p* = 0.0054; posterior horn PVWML rs = −0.4906, *p* = 0.0069; and parieto-occipital DWML rs = −0.3995, *p* = 0.0318), between the left medial temporal cortex and the PVWML in the left posterior horn (rs = −0.3739; *p* = 0.0457), between the right superior frontal cortex and three of four right WMLs (anterior horn PVWML rs = −0.4783, *p* = 0.0087; posterior horn PVWML rs = −0.4614, *p* = 0.0118; and parieto-occipital DWML rs = −0.4655, *p* = 0.0109), between the right middle frontal cortex and all of right four WMLs (anterior horn PVWML rs = −0.5468, *p* = 0.0021; posterior horn PVWML rs = −0.5279, *p* = 0.0032; frontal DWML rs = −0.4404, *p* = 0.0168; parieto-occipital DWML rs = −0.5010, *p* = 0.0056), and between the right medial frontal cortex and all of right four WMLs (anterior horn PVWML rs = −0.5557, *p* = 0.0018; posterior horn PVWML rs = −0.5240, *p* = 0.0035; frontal DWML rs = −0.4316, *p* = 0.0194; and parieto-occipital DWML rs = −0.5281, *p* = 0.0032). As for hallucination/delusion and abnormal activity, no significant correlations were observed between GMLs and WMLs.

When patients with AD-type dementia and aMCI were separately analyzed, hypoactivity showed a significant correlation between the left superior frontal cortex and the left frontal DWMLs in AD-type dementia (rs = 0.3305; *p* = 0.0348). In aMCI patients, hypoactivity showed significant correlations between the right middle, and medial frontal cortices and the right frontal DWMLs (rs = 0.6338, *p* = 0.0269, and rs = 0.5775, *p* = 0.0493, respectively). Hyperactivity showed significant correlations between the right superior frontal cortex and the PVWMLs in the right posterior horn and the right parieto-occipital DWMLs (rs = −0.7500, *p* = 0.0199, and rs = −0.7833, *p* = 0.0125, respectively), between the right middle frontal cortex and the PVWMLs in the right posterior horn and the right parieto-occipital DWMLs (rs = −0.7500, *p* = 0.0199, and rs = −0.7833, *p* = 0.0125, respectively), and between the right medial frontal cortex and the PVWMLs in the right posterior horn and the right parieto-occipital DWMLs (rs = −0.8500, *p* = 0.0037, and rs = −0.8667, *p* = 0.0025, respectively). For hallucination/delusion, appropriate statistical data could not be obtained.

All statistical data are presented in the Supplementary Tables.

## 4. Discussion

Our study demonstrated the critical correlation between GMLs and WMLs, which are involved in dementia symptoms and BPSD. Each domain of both cognitive impairment and BPSD did not always show a relationship with GMLs, whereas some domains showed a relationship with WMLs. Moreover, hyperactivity in BPSD showed a significant correlation between both sides of the frontal cortices and multiple WMLs.

WMLs can frequently be observed as high-intensity regions in brain MRI fluid-attenuated inversion recovery images of elderly patients [7]. The pathogenesis of WMLs has been reported to be influenced by the risk factors of atherosclerosis [17]. WMLs also play an important role in cognitive decline in patients with multiple sclerosis in which demyelination is exacerbated by inflammation [18]. Nevertheless, WMLs can be associated with declining cognitive function [19]. Of course, it is important to assess the damage to primary neurons to investigate the severity of dementia. Dementia patients may present various kinds of cognitive symptoms and BPSD during the disease course independent of the disease severity. It may be helpful to understand the pathomechanisms of these symptoms, including the relationship between GMLs and WMLs, for treatment and prevention.

In general, the left brain hemisphere is the dominant hemisphere of right-handed persons. Recent studies have revealed that a widespread network in the left cortical and subcortical area may contribute to the human language system [20]. Space recognition may be dominantly organized in the right hemisphere in right-handers [21]. According to the results of our logistic regression analysis, disturbed attention and memory showed correlations with the left frontal cortex. Disorientation and disorder of spatial perception showed a correlation with the right hemisphere. According to the results of our Spearman’s rank correlation coefficient analysis, the correlation between GMLs and WMLs was observed in both hemispheres in the analysis of disorientation, disturbed attention, memory disturbance, and disorder of spatial perception. These observations suggest that the evaluation of the relationship between GMLs and WMLs should be included in the analysis of cognitive impairment. Moreover, our data show that the relationship between the left precuneus and the PVWMLs in the left anterior and posterior horn was associated with disorientation, disturbed attention, memory disturbance, and disorder of spatial perception. The relationship between the right precuneus and the right DWMLs was also associated with disorientation and disturbed attention. Therefore, although the precuneus is one of the critical regions in AD pathology, it can be said that impaired connection between the precuneus and other regions in each hemisphere might influence dementia symptoms.

Moreover, according to the analysis of subcategories of BPSD, hypoactivity showed significant correlations between both sides of GMLs in the frontal cortex and DWMLs in the frontal lobe and between GMLs in the left frontal cortex and the PVWMLs in the left anterior horn. Hyperactivity showed a relationship between multiple GMLs in the frontal cortex and multiple WMLs in the deep and periventricular areas in both hemispheres. There might be a possibility that a functional imbalance between the right and left hemispheres influences the symptoms of BPSD. In fact, neuropsychiatric symptoms have been reported to show a significant relationship between focal gray matter atrophy and white matter hyperintensity lesions [22]. Delusion was reported to be related to the severity of GM-WM combined damage [14]. The emergence of hallucination was reported to be related to inappropriate organization of the right hemisphere against left frontal lobe activity [23]. Apathy and disinhibition have been supposed to be caused by the deficiency of salience network activity [24]. Unfortunately, because of the small number of patients, we could not analyze the GM-WM correlation in regard to hallucination/delusion, abnormal behavior, and disturbed circadian rhythm.

Another important finding in this study was obtained via the results of separate analyses of AD-type dementia and aMCI patients. Patients without AD-type dementia but with aMCI showed a significant correlation between the right precuneus and the right parieto-occipital DWMLs regarding symptoms of disorientation and memory disturbance. In the analysis of disturbed attention and disorder of spatial perception, patients with aMCI presented more correlations between GMLs and WMLs compared with those with AD-type dementia. Therefore, the role of GM-WM connectivity in dementia symptoms may possibly be changed depending on the different phases of the disease or the severity of the disease.

### Limitations

First, the number of participants in this study was small, due to our strict selection of enrolled patients within a period in which the conditions of imaging modalities and neuro-psychiatric tests remained unchanged. Therefore, while the accuracy of data may be reliable, there is potential for type 2 statistical error. Second, this is a retrospective study. All patients in this study were introduced from other clinics, so there may be a bias in the symptoms of patients. Third, we investigated the statistical relationship between GMLs and WMLs via multivariate analysis and did not analyze the functional connectivity between the two types of lesions. A larger prospective study in which functional connectivity can be assessed with other modalities, such as functional MRI or fractional anisotropy analysis, should be performed to confirm our findings in the future.

## 5. Conclusions

Correlation between GMLs and WMLs may be able to influence the manifestation of not only symptoms of cognitive impairment but also symptoms of BPSD. Especially, hyperactivity as a BPSD symptom may be caused by functional imbalances between both hemispheres of the frontal cortex and WMLs. Moreover, there is a possibility that the involvement of GMLs and WMLs may change depending on the disease phase or disease severity. This aspect will be verified in our findings using other modalities in the future.

## Figures and Tables

**Table 1:** Clinical characteristics of all patients.

	Total	AD Type	aMCI	*p*
*n*	156	102	54	
Male/female (*n*)	61/95	37/65	24/30	0.3198
Age (mean ± SD years old)	79.8 ± 7.4	80.7 ± 7.1	78.3 ± 7.7	0.0519
Hypertension	67.3%	70.6%	61.1%	0.2300
Hyperlipidemia	37.8%	36.3%	40.7%	0.5842
Diabetes mellitus	26.3%	31.4%	16.7%	0.0471
Heart diseases	10.9%	12.8%	7.4%	0.3088
MMSE-J (mean ± SD)	22.0 ± 4.1	20.4 ± 4.0	24.9 ± 2.4	<0.0001
Right-handedness	94.2%	90.2%	96.2%	0.1737
Cognitive impairments				
Disorientation (mean ± SD)	2.2 ± 2.1	2.9 ± 2.2	0.9 ± 1.1	<0.0001
Disturbed attention (mean ± SD)	2.3 ± 1.5	2.5 ± 1.5	1.9 ± 1.4	0.0158
Memory disturbance (mean ± SD)	2.1 ± 1.0	2.4 ± 0.9	1.7 ± 1.1	<0.0001
Impairment of language skill (mean ± SD)	0.2 ± 0.4	0.2 ± 0.4	0.1 ± 0.3	0.2997
Disorder of spatial perception (mean ± SD)	0.2 ± 0.4	0.2 ± 0.4	0.1 ± 0.3	0.0416
BPSD				
Hypoactivity	34.6%	44.1%	16.7%	0.0006
Hyperactivity	18.6%	20.6%	14.8%	0.3779
Hallucination/delusion	15.4%	21.6%	3.7%	0.0033
Abnormal behavior	7.1%	8.8%	3.7%	0.2347
Disturbed circadian rhythm	1.3%	2.0%	0%	0.3004

SD: standard deviation; AD type: Alzheimer’s-disease-type dementia; aMCI: amnestic mild cognitive impairment; MMSE-J: Mini-Mental State Examination Japanese version.

**Table 2:** Multivariate analysis of the involvement of atherosclerotic risks and WMLs.

Total	HT		HL		DM	
	Correlation Coefficient	*p*	Correlation Coefficient	*p*	Correlation Coefficient	*p*
L. anterior horn PVWML	0.2444	0.0021	0.0215	0.7897	0.1201	0.1353
R. anterior horn PVWML	0.2273	0.0043	0.0233	0.773	0.0797	0.3225
L. posterior horn PVWML	0.1334	0.097	0.0118	0.8833	0.105	0.1919
R. posterior horn PVWML	0.1283	0.1106	−0.091	0.2586	0.0718	0.3731
L. frontal DWML	0.0857	0.2876	0.0121	0.8807	0.0056	0.945
R. frontal DWML	0.1289	0.1089	0.0242	0.7642	0.0198	0.8058
L. parieto-occipital DWML	0.0567	0.4821	−0.0197	0.8068	0.0118	0.8837
R. parieto-occipital DWML	0.1102	0.1707	−0.0176	0.8276	0.0656	0.4161
AD type						
L. anterior horn PVWML	0.1645	0.0986	−0.0483	0.6296	0.1053	0.292
R. anterior horn PVWML	0.1543	0.1215	−0.0304	0.7618	0.0916	0.3601
L. posterior horn PVWML	0.05	0.618	−0.0464	0.6437	0.1047	0.2951
R. posterior horn PVWML	0.0286	0.7751	−0.1233	0.217	0.0812	0.4174
L. frontal DWML	−0.0143	0.8869	−0.1105	0.269	−0.0185	0.8538
R. frontal DWML	0.0664	0.5075	−0.065	0.5164	−0.0012	0.9906
L. parieto-occipital DWML	−0.0549	0.5833	−0.1177	0.2388	−0.0224	0.8232
R. parieto-occipital DWML	−0.012	0.9048	−0.1226	0.2196	0.0225	0.8222
aMCI						
L. anterior horn PVWML	0.4014	0.0029	0.1814	0.1937	0.1244	0.375
R. anterior horn PVWML	0.3788	0.0052	0.1768	0.2053	−0.0226	0.8721
L. posterior horn PVWML	0.3018	0.0281	0.1754	0.2091	0.0363	0.7962
R. posterior horn PVWML	0.3323	0.015	0.0162	0.9083	−0.0229	0.8705
L. frontal DWML	0.2632	0.0569	0.244	0.0783	0.0352	0.8023
R. frontal DWML	0.2631	0.057	0.2588	0.0613	0.0028	0.984
L. parieto-occipital DWML	0.2724	0.0485	0.2085	0.1341	0.0306	0.8279
R. parieto-occipital DWML	0.3619	0.0077	0.2455	0.0764	0.0987	0.4819

HT: hypertension; HL: hyperlipidemia; DM: diabetes mellitus; L.: left; R.: right; AD: Alzheimer’s dementia; aMCI: amnestic mild cognitive impairment; PVWML: periventricular white matter lesion; DWML: deep white matter lesion.

## Data Availability

The datasets used and analyzed in this study are available from the corresponding author on reasonable request.

## Supplementary Materials

The following supporting information can be downloaded at: https://www.mdpi.com/article/10.3390/jcm12247642/s1, Table S1: Logistic regression analysis for rhGM and WML in subcategories of cognitive impairment; Table S2: Logistic regression analysis for rhGM and WML in subcategories of BPSD; Table S3: Statistical data of Spearman’s Rank correlation coefficient for the association between rhGM and WML in each symptom of subcategories of cognitive impairment and BPSD; Table S4: Statistical data of Spearman’s Rank correlation coefficient for the connectivity between rhGM and WML in each symptom of subcategories of cognitive impairment in AD type dementia and aMCI; Table S5: Statistical data of Spearman’s Rank correlation coefficient for the connectivity between rhGM and WML in each symptom of subcategories of BPSD; Table S6: Statistical data of Spearman’s multivariate analysis for the connectivity between rhGM and WML in each symptom of subcategories of BPSD in AD type dementia and aMCI.
