# Supplementary material for: Association between Gray and White Matter Lesions and Its Involvement in Clinical Symptoms of Alzheimer’s-Type Dementia
Source: J Clin Med. 2023 Dec 12;12(24):7642. doi: 10.3390/jcm12247642 (PMC10744158; doi:10.3390/jcm12247642)
Supplement: Supplementary file 1 [file jcm-12-07642-s001.zip › jcm-2710485-supplementary.pdf]

Supplemental Table 1. Logistic regression analysis for rhGM and WML in subcategories of cognitive impairment.

Subcategories of cognitive impairment	Affected lesions	Estimated value of parameter (r)	p
**Disorientation**	L. anterior horn PVWML	1.51E-05	0.9352
	R. anterior horn PVWML	-0.0002451	0.1688
	L. posterior horn PVWML	0.00071261	0.2209
	R. posterior horn PVWML	0.00013688	0.7794
	L. frontal DWML	3.81E-05	0.8876
	R. frontal DWML	-1.8561e-6	0.9955
	L. parieto-occipital DWML	0.00014592	0.4732
	R. parieto-occipital DWML	-0.0002164	0.2553
	L. superior frontal	0.27602798	0.6994
	R. superior frontal	1.34439828	0.0736
	L. middle frontal	-0.6489697	0.1139
	R. middle frontal	0.02600387	0.9565
	L. medial frontal	-0.4051143	0.66
	R. medial frontal	-0.6636418	0.4969
	L. precuneus	-0.017222	0.8795
	R. precuneus	-0.0265759	0.9115
	L. medial temporal cortex	0.14533147	0.4865
	R. medial temporal cortex	-0.9594174	0.0010*
	L. posterior cingulate cortex	-0.0830059	0.7798
	R. posterior cingulate cortex	-0.1620319	0.6238
**Disturbed attention**	L. anterior horn PVWML	-0.0002258	0.2365
	R. anterior horn PVWML	7.44E-05	0.68
	L. posterior horn PVWML	-0.0011401	0.0566
	R. posterior horn PVWML	0.00105033	0.0378*
	L. frontal DWML	0.00014012	0.6093
	R. frontal DWML	-0.0002904	0.3917
	L. parieto-occipital DWML	0.00025673	0.2107
	R. parieto-occipital DWML	-5.0163e-5	0.794

	L. superior frontal	1.5468744	0.0370*
	R. superior frontal	-0.1504435	0.8485
	L. middle frontal	-1.4528986	0.0036*
	R. middle frontal	-0.2400132	0.6197
	L. medial frontal	-1.8434036	0.0546
	R. medial frontal	0.75081082	0.4625
	L. precuneus	0.37482484	0.3244
	R. precuneus	-0.0824114	0.8161
	L. medial temporal cortex	-0.0482112	0.8239
	R. medial temporal cortex	-0.2522661	0.3712
	L. posterior cingulate cortex	0.69335937	0.0456*
	R. posterior cingulate cortex	-0.5939853	0.0834
**Memory disturbance**	L. anterior horn PVWML	0.00016289	0.439
	R. anterior horn PVWML	-0.00024	0.231
	L. posterior horn PVWML	0.00114035	0.0769
	R. posterior horn PVWML	-0.0002294	0.6746
	L. frontal DWML	0.00013049	0.6734
	R. frontal DWML	-9.152e-5	0.8097
	L. parieto-occipital DWML	-1.1489e-5	0.9586
	R. parieto-occipital DWML	-0.0002665	0.221
	L. superior frontal	0.89242124	0.2616
	R. superior frontal	-0.6455645	0.4514
	L. middle frontal	-1.3584654	0.0232*
	R. middle frontal	0.33083971	0.5453
	L. medial frontal	-1.1364977	0.2598
	R. medial frontal	1.94375446	0.0751
	L. precuneus	0.21246599	0.6174
	R. precuneus	-0.3421685	0.3863
	L. medial temporal cortex	0.19761643	0.4058
	R. medial temporal cortex	-0.1653275	0.5894
	L. posterior cingulate cortex	0.33893861	0.3902
	R. posterior cingulate cortex	-0.3654343	0.327
**Disorder of spatial perception**	L. anterior horn PVWML	0.00049911	0.1146

	R. anterior horn PVWML	-0.0006988	0.0329*
	L. posterior horn PVWML	-0.0009039	0.3891
	R. posterior horn PVWML	0.00087654	0.3112
	L. frontal DWML	-0.0005233	0.2949
	R. frontal DWML	0.00087164	0.1707
	L. parieto-occipital DWML	0.0003555	0.3128
	R. parieto-occipital DWML	-0.0002183	0.4967
	L. superior frontal	-0.3580423	0.8354
	R. superior frontal	-0.0755377	0.9602
	L. middle frontal	-1.7902954	0.0963
	R. middle frontal	1.87557471	0.0490*
	L. medial frontal	3.02259961	0.1328
	R. medial frontal	-1.8966699	0.2607
	L. precuneus	-1.1513823	0.2126
	R. precuneus	-0.0512577	0.9506
	L. medial temporal cortex	0.31741918	0.4873
	R. medial temporal cortex	-0.636336	0.2771
	L. posterior cingulate cortex	1.34148984	0.0551
	R. posterior cingulate cortex	-0.5797976	0.4175
**Impairment of language skill**	L. anterior horn PVWML	0.00015812	0.6084
	R. anterior horn PVWML	-7.4247e-5	0.795
	L. posterior horn PVWML	0.00091324	0.4043
	R. posterior horn PVWML	-0.0008877	0.3143
	L. frontal DWML	-3.8388e-5	0.9344
	R. frontal DWML	-0.0004232	0.4265
	L. parieto-occipital DWML	-5.9538e-5	0.8685
	R. parieto-occipital DWML	0.00024063	0.466
	L. superior frontal	-1.4376762	0.3445
	R. superior frontal	-2.2509765	0.1519
	L. middle frontal	0.81891396	0.4035
	R. middle frontal	0.66620494	0.4842
	L. medial frontal	-0.5472886	0.7812
	R. medial frontal	1.8884173	0.3506
	L. precuneus	-0.1006141	0.8952

R. precuneus	0.49601942	0.5123
L. medial temporal cortex	0.3589806	0.4226
R. medial temporal cortex	0.04545698	0.9347
L. posterior cingulate cortex	0.16648874	0.8079
R. posterior cingulate cortex	-1.0539484	0.1444

L.: left; R.: right; PVWML: periventricular white matter lesion; DWML: deep white matter lesion.

Supplemental Table 2. Logistic regression analysis for rhGM and WML in subcategories of BPSD.

Subcategories of BPSD	Affected lesions	Estimated value of parameter	p
**Hypoactivity**	L. anterior horn PVWML	0.00013049	0.5792
	R. anterior horn PVWML	-0.0001627	0.4829
	L. posterior horn PVWML	-0.0007302	0.3216
	R. posterior horn PVWML	0.00021849	0.7247
	L. frontal DWML	-0.0004739	0.1949
	R. frontal DWML	0.00075456	0.1085
	L. parieto-occipital DWML	0.00035364	0.1919
	R. parieto-occipital DWML	-0.0001414	0.5628
	L. superior frontal	0.00274703	0.9976
	R. superior frontal	0.43532503	0.6459
	L. middle frontal	-0.340769	0.5719
	R. middle frontal	0.32132826	0.5942
	L. medial frontal	-0.5290616	0.6352
	R. medial frontal	0.40727337	0.7307
	L. precuneus	-0.219848	0.6258
	R. precuneus	0.05318266	0.8995
	L. medial temporal cortex	0.13669309	0.6036
	R. medial temporal cortex	-0.4460895	0.2184
	L. posterior cingulate cortex	0.74020356	0.094
	R. posterior cingulate cortex	-0.678032	0.0803
**Hyperactivity**	L. anterior horn PVWML	0.00032113	0.2937
	R. anterior horn PVWML	-8.634e-5	0.7434
	L. posterior horn PVWML	-0.0004951	0.5805
	R. posterior horn PVWML	-0.0001193	0.88
	L. frontal DWML	0.00038192	0.3532
	R. frontal DWML	-0.00072	0.1425
	L. parieto-occipital DWML	-0.0001055	0.7227

	R. parieto-occipital DWML	0.00016169	0.5756
	L. superior frontal	1.32581412	0.2756
	R. superior frontal	-1.2401227	0.3908
	L. middle frontal	0.17633868	0.8146
	R. middle frontal	0.44941357	0.5723
	L. medial frontal	-2.5940377	0.1013
	R. medial frontal	0.76139942	0.6585
	L. precuneus	-0.2331703	0.6824
	R. precuneus	0.73452328	0.2024
	L. medial temporal cortex	-0.0270732	0.9376
	R. medial temporal cortex	0.02688862	0.9517
	L. posterior cingulate cortex	0.30291191	0.5667
	R. posterior cingulate cortex	0.13421046	0.7855
**Hallucination/delusion**	L. anterior horn PVWML	7.65E-05	0.8223
	R. anterior horn PVWML	-0.0001746	0.5745
	L. posterior horn PVWML	0.00023802	0.8252
	R. posterior horn PVWML	0.00051725	0.5729
	L. frontal DWML	0.00141247	0.0298*
	R. frontal DWML	-0.0016071	0.0324*
	L. parieto-occipital DWML	-8.648e-5	0.8139
	R. parieto-occipital DWML	0.0000274	0.9396
	L. superior frontal	-0.8415228	0.5226
	R. superior frontal	2.28379644	0.108
	L. middle frontal	0.86765002	0.3351
	R. middle frontal	-0.1525485	0.8602
	L. medial frontal	0.80280377	0.6102
	R. medial frontal	-1.9970691	0.203
	L. precuneus	0.56412973	0.4048
	R. precuneus	-0.8307075	0.2148
	L. medial temporal cortex	-0.4825163	0.2684
	R. medial temporal cortex	-0.4576018	0.3866
	L. posterior cingulate cortex	0.36681488	0.5614
	R. posterior cingulate cortex	-0.1089585	0.8585
**Abnormal behavior**	L. anterior horn PVWML	5.27E-05	0.9155

R. anterior horn PVWML	-0.0003001	0.5149
L. posterior horn PVWML	0.00274921	0.1153
R. posterior horn PVWML	-0.0021141	0.0948
L. frontal DWML	-0.000707	0.2739
R. frontal DWML	0.00154307	0.0817
L. parieto-occipital DWML	-0.0003441	0.5064
R. parieto-occipital DWML	-0.0002113	0.6154
L. superior frontal	-1.5106336	0.5241
R. superior frontal	-1.3194548	0.5471
L. middle frontal	-1.3677095	0.3836
R. middle frontal	-0.1747298	0.8851
L. medial frontal	5.67414341	0.0461*
R. medial frontal	-1.3943382	0.5661
L. precuneus	-0.681001	0.5751
R. precuneus	-0.239504	0.86
L. medial temporal cortex	0.95776964	0.0962
R. medial temporal cortex	1.06097187	0.1472
L. posterior cingulate cortex	-1.3593349	0.2151
R. posterior cingulate cortex	-0.021799	0.9786

BPSD: behavioral and psychological symptoms of dementia; L.: left; R.: right; PVWML: periventricular white matter lesion; DWML: deep white matter lesion.

Supplemental Table 3. Statistical data of Spearman's Rank correlation coefficient for the association between rhGM and WML in each symptom of subcategories of cognitive impairment and BPSD.

		Left		Right	
**rhGM**	**WML**	**rs**	**p**	**rs**	**p**
**Disorientation**					
Superior frontal	Anterior horn PVWML	0.0079	0.9339	0.01	0.9163
	Posterior horn PVWML	-0.0886	0.3484	-0.0730	0.4403
	Frontal DWML	0.053	0.5751	0.0376	0.6913
	Parieto-occipital DWML	-0.0329	0.7278	-0.0857	0.3647
Middle frontal	Anterior horn PVWML	0.0502	0.5958	0.0588	0.5346
	Posterior horn PVWML	-0.0862	0.3616	-0.0381	0.687
	Frontal DWML	0.089	0.3462	0.0846	0.371
	Parieto-occipital DWML	-0.0574	0.5444	-0.0836	0.3765
Medial frontal	Anterior horn PVWML	0.0003	0.9973	-0.0151	0.8734
	Posterior horn PVWML	-0.1053	0.2648	-0.1022	0.2794
	Frontal DWML	0.0357	0.7063	0.0048	0.9599
	Parieto-occipital DWML	-0.0376	0.6913	-0.1228	0.1929
Precuneus	Anterior horn PVWML	-0.1967	0.0360*	-0.1481	0.1157
	Posterior horn PVWML	-0.2060	0.0279*	-0.1502	0.1107
	Frontal DWML	-0.0942	0.3186	-0.1612	0.0866
	Parieto-occipital DWML	-0.1333	0.1574	-0.1993	0.0336*
Medial temporal	Anterior horn PVWML	0.0463	0.6244	0.1129	0.2319
	Posterior horn PVWML	-0.0309	0.7444	-0.0032	0.973
	Frontal DWML	0.1063	0.2601	0.1264	0.1803
	Parieto-occipital DWML	0.0413	0.6627	-0.0160	0.8656
PCC	Anterior horn PVWML	-0.0768	0.417	-0.0297	0.7538
	Posterior horn PVWML	-0.0862	0.362	0.0295	0.7555
	Frontal DWML	-0.0400	0.6726	0.0118	0.9012
	Parieto-occipital DWML	-0.1214	0.1981	-0.0517	0.5848
**Disturbed attention**					
Superior frontal	Anterior horn PVWML	-0.0265	0.7665	-0.0197	0.8253
	Posterior horn PVWML	-0.1350	0.1286	-0.1367	0.1238

	Frontal DWML	0.0082	0.9264	0.0011	0.9898
	Parieto-occipital DWML	-0.0945	0.2886	-0.1356	0.127
Middle frontal	Anterior horn PVWML	0.0244	0.7842	0.0349	0.6954
	Posterior horn PVWML	-0.1274	0.1517	-0.0961	0.2806
	Frontal DWML	0.0345	0.6987	0.0481	0.5895
	Parieto-occipital DWML	-0.1086	0.2223	-0.1168	0.1893
Medial frontal	Anterior horn PVWML	-0.0381	0.6695	-0.0328	0.7134
	Posterior horn PVWML	-0.1433	0.1065	-0.1456	0.1009
	Frontal DWML	-0.0106	0.9053	-0.0159	0.8589
	Parieto-occipital DWML	-0.1006	0.2585	-0.1495	0.0921
Precuneus	Anterior horn PVWML	-0.2349	0.0076*	-0.1713	0.0532
	Posterior horn PVWML	-0.2191	0.0129*	-0.1612	0.0691
	Frontal DWML	-0.1912	0.0306*	-0.2221	0.0117*
	Parieto-occipital DWML	-0.2199	0.0126*	-0.2129	0.0158*
Medial temporal	Anterior horn PVWML	0.0423	0.6354	0.1549	0.0808
	Posterior horn PVWML	-0.0644	0.4699	0.0393	0.6599
	Frontal DWML	0.063	0.48	0.1323	0.1365
	Parieto-occipital DWML	-0.0020	0.9819	0.021	0.8142
PCC	Anterior horn PVWML	-0.0198	0.8247	0.0333	0.7091
	Posterior horn PVWML	-0.0445	0.618	0.0283	0.7512
	Frontal DWML	-0.0358	0.6881	0.0365	0.6826
	Parieto-occipital DWML	-0.1141	0.1996	-0.0508	0.5691
**Memory disturbance**					
Superior frontal	Anterior horn PVWML	0.0188	0.8284	-0.0044	0.959
	Posterior horn PVWML	-0.1103	0.2011	-0.1143	0.1851
	Frontal DWML	0.0684	0.4285	0.0755	0.3824
	Parieto-occipital DWML	-0.0461	0.5941	-0.0822	0.3413
Middle frontal	Anterior horn PVWML	0.0354	0.6825	0.0352	0.6842
	Posterior horn PVWML	-0.1415	0.1004	-0.0818	0.3436
	Frontal DWML	0.0647	0.4542	0.0982	0.2553
	Parieto-occipital DWML	-0.0990	0.2516	-0.0868	0.3148
Medial frontal	Anterior horn PVWML	0.0194	0.8225	-0.0133	0.8782
	Posterior horn PVWML	-0.1218	0.1578	-0.1305	0.1301
	Frontal DWML	0.0588	0.4964	0.0445	0.6068

	Parieto-occipital DWML	-0.0576	0.5053	-0.1182	0.1706
Precuneus	Anterior horn PVWML	-0.1893	0.0273*	-0.1725	0.0446*
	Posterior horn PVWML	-0.1762	0.0401*	-0.1317	0.1263
	Frontal DWML	-0.1013	0.2405	-0.1393	0.1058
	Parieto-occipital DWML	-0.1424	0.0981	-0.1655	0.0542
Medial temporal	Anterior horn PVWML	0.053	0.5396	0.1412	0.101
	Posterior horn PVWML	-0.0445	0.6069	0.0359	0.6783
	Frontal DWML	0.114	0.1863	0.1756	0.0409*
	Parieto-occipital DWML	0.0263	0.7616	0.027	0.755
PCC	Anterior horn PVWML	-0.0396	0.6474	0.0629	0.4666
	Posterior horn PVWML	-0.0708	0.4127	0.0417	0.6298
	Frontal DWML	0.0022	0.9799	0.1231	0.1535
	Parieto-occipital DWML	-0.1060	0.2194	-0.0102	0.9058
**Disorder of spatial perception**					
Superior frontal	Anterior horn PVWML	0.0702	0.7279	0.1713	0.393
	Posterior horn PVWML	-0.1838	0.3589	-0.1880	0.3476
	Frontal DWML	0.0397	0.844	0.1098	0.5858
	Parieto-occipital DWML	-0.0085	0.9663	0.0824	0.6828
Middle frontal	Anterior horn PVWML	-0.0043	0.9831	0.1929	0.3349
	Posterior horn PVWML	-0.2063	0.3018	-0.1258	0.5319
	Frontal DWML	-0.0367	0.8558	0.1602	0.4248
	Parieto-occipital DWML	-0.0324	0.8727	0.0812	0.6872
Medial frontal	Anterior horn PVWML	0.0391	0.8466	0.1075	0.5937
	Posterior horn PVWML	-0.1935	0.3334	-0.3199	0.1038
	Frontal DWML	0.0043	0.9831	0.0141	0.9445
	Parieto-occipital DWML	-0.0128	0.9494	-0.0501	0.8042
Precuneus	Anterior horn PVWML	-0.4231	0.0279*	-0.2162	0.2789
	Posterior horn PVWML	-0.2918	0.1397	-0.2821	0.1541
	Frontal DWML	-0.2293	0.25	-0.2284	0.2519
	Parieto-occipital DWML	-0.1630	0.4166	-0.1032	0.6086
Medial temporal	Anterior horn PVWML	-0.1233	0.5399	0.152	0.449
	Posterior horn PVWML	-0.2409	0.2261	-0.1856	0.354
	Frontal DWML	-0.0590	0.77	0.0752	0.7093
	Parieto-occipital DWML	-0.0540	0.7889	0.0769	0.7029

PCC	Anterior horn PVWML	-0.1001	0.6193	-0.0110	0.9566
	Posterior horn PVWML	-0.2155	0.2803	-0.2405	0.2268
	Frontal DWML	-0.1098	0.5858	-0.0997	0.6209
	Parieto-occipital DWML	-0.1667	0.406	-0.1465	0.4658
**Impairment of language skill**					
Superior frontal	Anterior horn PVWML	-0.0530	0.8056	0.0435	0.8401
	Posterior horn PVWML	0.02	0.9261	0.0339	0.875
	Frontal DWML	0.0431	0.8414	0.1654	0.44
	Parieto-occipital DWML	-0.0400	0.8528	-0.0765	0.7223
Middle frontal	Anterior horn PVWML	-0.0052	0.9807	0.1087	0.6132
	Posterior horn PVWML	-0.0383	0.8591	0.0922	0.6684
	Frontal DWML	0.0662	0.7585	0.2097	0.3253
	Parieto-occipital DWML	-0.0922	0.6684	-0.0122	0.955
Medial frontal	Anterior horn PVWML	-0.0070	0.9743	0.1035	0.6304
	Posterior horn PVWML	-0.0130	0.9518	0.0739	0.7314
	Frontal DWML	0.068	0.7523	0.2463	0.246
	Parieto-occipital DWML	-0.0609	0.7775	-0.0191	0.9293
Precuneus	Anterior horn PVWML	-0.2800	0.1851	-0.2391	0.2604
	Posterior horn PVWML	-0.1096	0.6103	-0.1896	0.375
	Frontal DWML	-0.1983	0.3531	-0.1984	0.3526
	Parieto-occipital DWML	-0.2061	0.334	-0.3400	0.104
Medial temporal	Anterior horn PVWML	0.2217	0.2977	0.4165	0.0429*
	Posterior horn PVWML	0.1539	0.4727	0.3078	0.1434
	Frontal DWML	0.1743	0.4153	0.3246	0.1217
	Parieto-occipital DWML	0.1183	0.5821	0.1878	0.3795
PCC	Anterior horn PVWML	-0.1635	0.4453	0.1374	0.522
	Posterior horn PVWML	-0.2783	0.188	0.04	0.8528
	Frontal DWML	-0.2192	0.3035	0.1158	0.5902
	Parieto-occipital DWML	-0.3939	0.0568	-0.0217	0.9197

rhGM: regional hypoperfusion of gray matter; PVWML: periventricular white matter lesion; DWML: deep white matter lesion; PCC: posterior cingulate cortex.

Supplemental Table 4. Statistical data of Spearman's Rank correlation coefficient for the connectivity between rhGM and WML in each symptom of subcategories of cognitive impairment in AD type dementia and aMCI.

		AD type				aMCI			
		Left		Right		Left		Right	
rhGM	WML	rs	p	rs	p	rs	p	rs	p
**Disorientation**									
Superior frontal	Anterior horn PVWML	0.0251	0.8195	0.0299	0.7859	-0.0394	0.8392	-0.0951	0.6237
	Posterior horn PVWML	-0.0865	0.4314	-0.0586	0.594	-0.1424	0.4613	-0.1453	0.452
	Frontal DWML	0.0553	0.6151	0.0327	0.7666	0.0315	0.871	0.0022	0.9909
	Parieto-occipital DWML	-0.0248	0.8215	-0.0299	0.7858	-0.0345	0.8591	-0.2417	0.2066
Middle frontal	Anterior horn PVWML	0.0277	0.8014	0.0467	0.6712	0.0586	0.7626	0.065	0.7375
	Posterior horn PVWML	-0.1135	0.3008	-0.0247	0.8224	-0.0621	0.7491	-0.0143	0.9414
	Frontal DWML	0.0496	0.6523	0.0403	0.7145	0.1527	0.429	0.2026	0.2919
	Parieto-occipital DWML	-0.0649	0.5549	-0.0686	0.5329	-0.0281	0.885	-0.0926	0.6327
Medial frontal	Anterior horn PVWML	-0.0049	0.9645	-0.0352	0.7493	-0.0414	0.8312	-0.0266	0.8911
	Posterior horn PVWML	-0.1239	0.2586	-0.1229	0.2623	-0.1384	0.4739	-0.0714	0.7127
	Frontal DWML	0.0138	0.9001	-0.0423	0.7004	0.0318	0.87	0.0918	0.6359
	Parieto-occipital DWML	-0.0518	0.6376	-0.1242	0.2575	-0.0404	0.8352	-0.1579	0.4133
Precuneus	Anterior horn PVWML	-0.1384	0.2066	-0.1107	0.313	-0.3458	0.0661	-0.2911	0.1255
	Posterior horn PVWML	-0.1759	0.1073	-0.1262	0.2498	-0.3409	0.0704	-0.2882	0.1295
	Frontal DWML	-0.0149	0.8922	-0.1453	0.1845	-0.2722	0.1531	-0.2469	0.1967

	Parieto-occipital DWML	-0.0573	0.6027	-0.1204	0.2723	-0.3079	0.1042	-0.4924	0.0067*
Medial temporal	Anterior horn PVWML	-0.0037	0.9734	0.1112	0.3109	0.1768	0.3588	0.1414	0.4644
	Posterior horn PVWML	-0.0225	0.838	0.0574	0.6017	-0.0690	0.7222	-0.0847	0.6621
	Frontal DWML	0.0343	0.7554	0.09	0.4127	0.318	0.0927	0.2751	0.1487
	Parieto-occipital DWML	0.0605	0.5824	0.0419	0.7034	0.0049	0.9798	-0.1175	0.5438
PCC	Anterior horn PVWML	-0.1066	0.3314	-0.0758	0.4907	-0.0369	0.8491	0.0847	0.6621
	Posterior horn PVWML	-0.0826	0.4525	0.0059	0.9573	-0.1906	0.3219	0.1094	0.5723
	Frontal DWML	-0.0719	0.5133	-0.0768	0.4847	0.0325	0.867	0.2209	0.2495
	Parieto-occipital DWML	-0.1004	0.3603	-0.0487	0.6577	-0.1734	0.3684	-0.0943	0.6264
**Disturbed attention**									
Superior frontal	Anterior horn PVWML	-0.0084	0.9383	-0.0008	0.9943	-0.0695	0.6658	-0.1213	0.4498
	Posterior horn PVWML	-0.1254	0.2471	-0.0905	0.4047	-0.2392	0.132	-0.2732	0.084
	Frontal DWML	0.0112	0.9177	-0.0235	0.829	-0.0464	0.7735	-0.0318	0.8436
	Parieto-occipital DWML	-0.0617	0.5705	-0.0576	0.5964	-0.1918	0.2296	-0.3238	0.0389*
Middle frontal	Anterior horn PVWML	0.0238	0.827	0.02	0.854	0.0152	0.9251	0.0114	0.9435
	Posterior horn PVWML	-0.1415	0.1912	-0.0794	0.4648	-0.1510	0.3458	-0.1303	0.4167
	Frontal DWML	0.0216	0.8423	-0.0164	0.8798	0.0067	0.9668	0.107	0.5056
	Parieto-occipital DWML	-0.0892	0.4113	-0.0894	0.4101	-0.1747	0.2745	-0.1851	0.2466
Medial frontal	Anterior horn PVWML	-0.0256	0.8139	-0.0557	0.6086	-0.1026	0.5232	-0.0620	0.7001

	Posterior horn PVWML	-0.1316	0.2243	-0.1406	0.1939	-0.2674	0.091	-0.2148	0.1774
	Frontal DWML	-0.0100	0.9264	-0.0781	0.472	-0.0998	0.5348	-0.0021	0.9896
	Parieto-occipital DWML	-0.0679	0.5318	-0.1205	0.2663	-0.2420	0.1274	-0.2721	0.0853
Precuneus	Anterior horn PVWML	-0.2136	0.0469*	-0.1940	0.0718	-0.2805	0.0757	-0.1621	0.3112
	Posterior horn PVWML	-0.2179	0.0427*	-0.1618	0.1343	-0.2843	0.0716	-0.2169	0.1731
	Frontal DWML	-0.1136	0.2948	-0.2192	0.0414*	-0.3711	0.0169*	-0.3230	0.0394*
	Parieto-occipital DWML	-0.1069	0.3242	-0.1213	0.2632	-0.4870	0.0012*	-0.4546	0.0028*
Medial temporal	Anterior horn PVWML	-0.0040	0.9705	0.1124	0.3	0.1085	0.4997	0.1827	0.253
	Posterior horn PVWML	-0.0238	0.8266	0.0368	0.7349	-0.1928	0.2272	0.0334	0.8359
	Frontal DWML	0.0139	0.898	0.0634	0.5598	0.1116	0.4871	0.1727	0.2803
	Parieto-occipital DWML	0.0578	0.5946	0.0411	0.7051	-0.1662	0.299	-0.0576	0.7204
PCC	Anterior horn PVWML	-0.0292	0.7884	-0.0059	0.9564	-0.0249	0.8771	0.0392	0.8077
	Posterior horn PVWML	-0.0542	0.6178	0.0204	0.8514	-0.0904	0.574	-0.0124	0.9388
	Frontal DWML	-0.0390	0.7198	-0.0423	0.6972	-0.0449	0.7805	0.086	0.5928
	Parieto-occipital DWML	-0.0631	0.5615	-0.0185	0.8652	-0.2437	0.1246	-0.1917	0.2298
**Memory disturbance**									
Superior frontal	Anterior horn PVWML	0.004	0.9694	-0.0099	0.9252	0.0894	0.5686	-0.0187	0.9051
	Posterior horn PVWML	-0.1324	0.2057	-0.1056	0.3136	-0.0811	0.6052	-0.1558	0.3183
	Frontal DWML	0.0485	0.6441	0.0357	0.7341	0.1275	0.4153	0.109	0.4864

	Parieto-occipital DWML	-0.0660	0.5298	-0.0406	0.6992	0.0145	0.9265	-0.1855	0.2336
Middle frontal	Anterior horn PVWML	0.0157	0.8815	0.0277	0.7923	0.0951	0.5439	0.0523	0.7393
	Posterior horn PVWML	-0.1754	0.0926	-0.0708	0.4999	-0.0905	0.564	-0.0796	0.612
	Frontal DWML	0.0351	0.7381	0.0519	0.6211	0.1237	0.4294	0.1707	0.2737
	Parieto-occipital DWML	-0.1236	0.2378	-0.0659	0.5302	-0.0497	0.7517	-0.1198	0.4443
Medial frontal	Anterior horn PVWML	-0.0091	0.9311	-0.0576	0.5835	0.0885	0.5726	0.0329	0.834
	Posterior horn PVWML	-0.1569	0.1331	-0.1546	0.139	-0.0741	0.6365	-0.1102	0.4816
	Frontal DWML	0.0183	0.8617	-0.0274	0.7945	0.1278	0.4142	0.1274	0.4155
	Parieto-occipital DWML	-0.0876	0.4035	-0.1208	0.2488	-0.0036	0.9816	-0.1454	0.3521
Precuneus	Anterior horn PVWML	-0.1655	0.1129	-0.1522	0.1452	-0.2152	0.1658	-0.2481	0.1087
	Posterior horn PVWML	-0.1672	0.1091	-0.1122	0.2843	-0.2064	0.1841	-0.2256	0.1458
	Frontal DWML	-0.0239	0.8204	-0.1233	0.239	-0.2260	0.1451	-0.2272	0.1429
	Parieto-occipital DWML	-0.0599	0.5687	-0.0741	0.4802	-0.2912	0.0582	-0.4036	0.0073*
Medial temporal	Anterior horn PVWML	-0.0235	0.8234	0.1101	0.2934	0.1868	0.2303	0.1982	0.2026
	Posterior horn PVWML	-0.0339	0.7469	0.0338	0.7479	-0.1041	0.5067	0.0686	0.6622
	Frontal DWML	0.0335	0.7501	0.1225	0.2421	0.2507	0.1049	0.2731	0.0764
	Parieto-occipital DWML	0.0314	0.7649	0.0505	0.6305	-0.0116	0.9414	-0.0055	0.9722
PCC	Anterior horn PVWML	-0.0675	0.52	-0.0180	0.8637	-0.0040	0.9797	0.1732	0.2667

	Posterior horn PVWML	-0.0864	0.4101	0.0105	0.9204	-0.0893	0.5693	0.0856	0.5851
	Frontal DWML	-0.0265	0.8012	0.0143	0.8916	0.0599	0.7029	0.2797	0.0693
	Parieto-occipital DWML	-0.1016	0.3323	-0.0003	0.9978	-0.1369	0.3814	-0.0873	0.5778
**Disorder of spatial perception**									
Superior frontal	Anterior horn PVWML	0.0909	0.6951	0.0805	0.7286	0.0857	0.8717	-0.0290	0.9565
	Posterior horn PVWML	-0.1610	0.4856	-0.1584	0.4927	-0.7714	0.0724	-0.3143	0.5441
	Frontal DWML	0.0371	0.873	0.0806	0.7283	-0.5429	0.2657	-0.5429	0.2657
	Parieto-occipital DWML	-0.0078	0.9733	0.113	0.6258	-0.8857	0.0188*	-0.7714	0.0724
Middle frontal	Anterior horn PVWML	0.1117	0.6298	0.1143	0.6218	-0.2571	0.6228	-0.2609	0.6175
	Posterior horn PVWML	-0.1844	0.4236	-0.1039	0.654	0.1429	0.7872	-0.1429	0.7872
	Frontal DWML	0.0241	0.9174	0.156	0.4994	-0.4286	0.3965	-0.7143	0.1108
	Parieto-occipital DWML	-0.0195	0.9332	0.1078	0.6419	-0.0857	0.8717	-0.7714	0.0724
Medial frontal	Anterior horn PVWML	0.0675	0.7712	-0.0312	0.8933	0.1429	0.7872	0.1449	0.7841
	Posterior horn PVWML	-0.2260	0.3246	-0.3338	0.1392	-0.6000	0.208	-0.5429	0.2657
	Frontal DWML	-0.0235	0.9196	-0.0949	0.6823	-0.6571	0.1562	-0.3143	0.5441
	Parieto-occipital DWML	-0.0883	0.7035	-0.1013	0.6622	-0.7714	0.0724	-0.7143	0.1108
Precuneus	Anterior horn PVWML	-0.4260	0.0542	-0.3052	0.1785	-0.4286	0.3965	0.1449	0.7841
	Posterior horn PVWML	-0.4234	0.0558	-0.2455	0.2835	-0.3714	0.4685	-0.5429	0.2657
	Frontal DWML	-0.3029	0.1819	-0.2120	0.3563	-0.0857	0.8717	-0.3143	0.5441

	Parieto-occipital DWML	-0.2416	0.2915	-0.0532	0.8187	-0.4286	0.3965	-0.7143	0.1108
Medial temporal	Anterior horn PVWML	-0.2208	0.3362	0.0922	0.691	0.2609	0.6175	0.2319	0.6584
	Posterior horn PVWML	-0.1857	0.4203	-0.1974	0.3911	-0.8117	0.0499*	-0.0857	0.8717
	Frontal DWML	-0.0580	0.8029	0.0871	0.7073	-0.5508	0.2574	-0.2571	0.6228
	Parieto-occipital DWML	-0.0026	0.9911	0.0974	0.6745	-0.8986	0.0149*	-0.2571	0.6228
PCC	Anterior horn PVWML	-0.1247	0.5903	-0.1013	0.6622	0.2571	0.6228	-0.2319	0.6584
	Posterior horn PVWML	-0.2883	0.205	-0.2545	0.2655	0.1429	0.7872	-0.1429	0.7872
	Frontal DWML	-0.0945	0.6838	-0.1665	0.4708	-0.4857	0.3287	-0.5429	0.2657
	Parieto-occipital DWML	-0.2948	0.1945	-0.1753	0.4472	-0.0286	0.9572	-0.8857	0.0188*
**Impairment of language skill**									
Superior frontal	Anterior horn PVWML	-0.1123	0.6472	-0.0632	0.7973	0.7	0.1881	1	<.0001*
	Posterior horn PVWML	-0.0491	0.8417	0.0439	0.8585	0.7	0.1881	0.6	0.2848
	Frontal DWML	0.0193	0.9374	0.115	0.6393	0.9	0.0374*	0.9	0.0374*
	Parieto-occipital DWML	-0.0509	0.8361	-0.0649	0.7918	0.4	0.5046	0.7	0.1881
Middle frontal	Anterior horn PVWML	-0.0632	0.7973	0.0193	0.9375	0.7	0.1881	0.9	0.0374*
	Posterior horn PVWML	-0.0860	0.7264	0.1211	0.6215	0.7	0.1881	0.7	0.1881
	Frontal DWML	0.0123	0.9601	0.1632	0.5043	0.9	0.0374*	1	<.0001*
	Parieto-occipital DWML	-0.1123	0.6472	0.0246	0.9205	0.4	0.5046	0.9	0.0374*
Medial frontal	Anterior horn PVWML	-0.1193	0.6266	-0.0404	0.8697	0.7	0.1881	1	<.0001*

	Posterior horn PVWML	-0.0754	0.7589	0.0807	0.7426	0.7	0.1881	0.6	0.2848
	Frontal DWML	-0.0404	0.8695	0.1764	0.4701	0.9	0.0374*	0.9	0.0374*
	Parieto-occipital DWML	-0.1018	0.6785	-0.0526	0.8306	0.4	0.5046	0.7	0.1881
Precuneus	Anterior horn PVWML	-0.2070	0.3951	-0.2316	0.3401	-0.7000	0.1881	-0.6000	0.2848
	Posterior horn PVWML	-0.0842	0.7318	-0.1368	0.5764	-0.2000	0.7471	-0.2000	0.7471
	Frontal DWML	-0.0703	0.7749	-0.1659	0.4974	-0.6000	0.2848	-0.7000	0.1881
	Parieto-occipital DWML	-0.1158	0.6369	-0.2333	0.3364	-0.1000	0.8729	-0.5000	0.391
Medial temporal	Anterior horn PVWML	0.0912	0.7103	0.3158	0.1878	0.9	0.0374*	1	<.0001*
	Posterior horn PVWML	0.0895	0.7157	0.3158	0.1878	0.6	0.2848	0.6	0.2848
	Frontal DWML	-0.0105	0.9658	0.1913	0.4327	1	<.0001*	0.9	0.0374*
	Parieto-occipital DWML	0.0807	0.7426	0.1228	0.6165	0.3	0.6238	0.7	0.1881
PCC	Anterior horn PVWML	-0.1930	0.4286	-0.0596	0.8083	0.1	0.8729	0.9	0.0374*
	Posterior horn PVWML	-0.2719	0.2601	-0.0702	0.7753	-0.1000	0.8729	0.7	0.1881
	Frontal DWML	-0.2794	0.2466	-0.1176	0.6316	0.3	0.6238	1	<.0001*
	Parieto-occipital DWML	-0.3930	0.096	-0.1982	0.4159	-0.5000	0.391	0.9	0.0374*

rhGM: regional hypoperfusion of gray matter; PVWML: periventricular white matter lesion; DWML: deep white matter lesion; PCC: posterior cingulate cortex.

Supplemental Table 5. Statistical data of Spearman's Rank correlation coefficient for the connectivity between rhGM and WML in each symptom of subcategories of BPSD.

		Left		Right	
**rhGM**	**WML**	**rs**	**p**	rs	p
**Hypoactivity**					
Superior frontal	Anterior horn PVWML	0.3079	0.0249*	0.2503	0.0707
	Posterior horn PVWML	0.1682	0.2287	0.0081	0.9538
	Frontal DWML	0.3684	0.0066*	0.301	0.0285*
	Parieto-occipital DWML	0.3141	0.0220*	0.1345	0.337
Middle frontal	Anterior horn PVWML	0.2976	0.0304*	0.2378	0.0864
	Posterior horn PVWML	0.0983	0.484	-0.0024	0.9865
	Frontal DWML	0.3553	0.0090*	0.3065	0.0256*
	Parieto-occipital DWML	0.216	0.1204	0.0868	0.5368
Medial frontal	Anterior horn PVWML	0.282	0.0408*	0.2535	0.067
	Posterior horn PVWML	0.1161	0.4077	0.0413	0.7689
	Frontal DWML	0.3368	0.0137*	0.3181	0.0203*
	Parieto-occipital DWML	0.2673	0.053	0.1256	0.3701
Precuneus	Anterior horn PVWML	0.0252	0.8576	0.0822	0.5582
	Posterior horn PVWML	-0.0345	0.8062	-0.0323	0.8184
	Frontal DWML	0.11	0.4329	0.0468	0.7395
	Parieto-occipital DWML	0.0812	0.5633	0.0083	0.9529
Medial temporal	Anterior horn PVWML	0.2663	0.0539	0.181	0.1946
	Posterior horn PVWML	0.1427	0.3079	-0.0559	0.6908
	Frontal DWML	0.3023	0.0278*	0.2131	0.1256
	Parieto-occipital DWML	0.2496	0.0715	0.0295	0.8338
PCC	Anterior horn PVWML	0.1424	0.3091	0.1605	0.2511
	Posterior horn PVWML	0.0839	0.5505	0.1111	0.4283
	Frontal DWML	0.1573	0.2607	0.1929	0.1664
	Parieto-occipital DWML	0.0724	0.6062	0.1459	0.2971
**Hyperactivity**					
Superior frontal	Anterior horn PVWML	-0.4044	0.0295*	-0.4783	0.0087*
	Posterior horn PVWML	-0.3611	0.0543	-0.4614	0.0118*
	Frontal DWML	-0.1123	0.5618	-0.3351	0.0755
	Parieto-occipital DWML	-0.2951	0.1202	-0.4655	0.0109*

Middle frontal	Anterior horn PVWML	-0.5197	0.0039*	-0.5468	0.0021*
	Posterior horn PVWML	-0.5158	0.0042*	-0.5279	0.0032*
	Frontal DWML	-0.2118	0.2699	-0.4404	0.0168*
	Parieto-occipital DWML	-0.4039	0.0298*	-0.5010	0.0056*
Medial frontal	Anterior horn PVWML	-0.5030	0.0054*	-0.5557	0.0018*
	Posterior horn PVWML	-0.4906	0.0069*	-0.5240	0.0035*
	Frontal DWML	-0.2540	0.1837	-0.4316	0.0194*
	Parieto-occipital DWML	-0.3995	0.0318*	-0.5281	0.0032*
Precuneus	Anterior horn PVWML	-0.2901	0.1268	-0.1739	0.367
	Posterior horn PVWML	-0.1862	0.3335	-0.1165	0.5472
	Frontal DWML	-0.0672	0.7289	-0.3154	0.0956
	Parieto-occipital DWML	-0.2635	0.1672	-0.1980	0.3031
Medial temporal	Anterior horn PVWML	-0.2645	0.1655	-0.3251	0.0853
	Posterior horn PVWML	-0.3739	0.0457*	-0.3665	0.0505
	Frontal DWML	-0.0640	0.7413	-0.1714	0.374
	Parieto-occipital DWML	-0.1941	0.313	-0.3241	0.0863
PCC	Anterior horn PVWML	-0.2833	0.1365	-0.1562	0.4186
	Posterior horn PVWML	-0.2355	0.2188	-0.1527	0.429
	Frontal DWML	-0.0010	0.996	-0.0081	0.9666
	Parieto-occipital DWML	-0.2030	0.291	-0.1177	0.543
**Hallucination/delusion**					
Superior frontal	Anterior horn PVWML	0.0949	0.6668	-0.0949	0.6668
	Posterior horn PVWML	0.0899	0.6833	-0.1265	0.5652
	Frontal DWML	0.2264	0.299	0.0663	0.7638
	Parieto-occipital DWML	0.2352	0.28	-0.0899	0.6833
Middle frontal	Anterior horn PVWML	-0.0306	0.8896	-0.0237	0.9145
	Posterior horn PVWML	-0.0652	0.7675	0.0277	0.9003
	Frontal DWML	0.0728	0.7413	0.095	0.6665
	Parieto-occipital DWML	0.001	0.9964	-0.0375	0.8649
Medial frontal	Anterior horn PVWML	-0.0010	0.9964	-0.1858	0.3961
	Posterior horn PVWML	-0.0682	0.7572	-0.1255	0.5683
	Frontal DWML	0.1164	0.5969	0	1
	Parieto-occipital DWML	0.1403	0.5231	-0.1018	0.644
Precuneus	Anterior horn PVWML	-0.0573	0.7951	-0.1294	0.5561

	Posterior horn PVWML	-0.0306	0.8896	0.0988	0.6537
	Frontal DWML	0.0867	0.6941	-0.0544	0.8053
	Parieto-occipital DWML	0.1018	0.644	0.0296	0.8932
Medial temporal	Anterior horn PVWML	0.1038	0.6376	0.0771	0.7266
	Posterior horn PVWML	0.2915	0.1772	0.2342	0.282
	Frontal DWML	0.369	0.0831	0.3116	0.1477
	Parieto-occipital DWML	0.4081	0.0532	0.1591	0.4683
PCC	Anterior horn PVWML	-0.2115	0.3327	-0.2125	0.3304
	Posterior horn PVWML	-0.0652	0.7675	-0.0079	0.9714
	Frontal DWML	-0.0124	0.9553	0.0208	0.9251
	Parieto-occipital DWML	-0.0010	0.9964	-0.0237	0.9145
**Abnormal behavior**					
Superior frontal	Anterior horn PVWML	-0.1000	0.7699	-0.2000	0.5554
	Posterior horn PVWML	-0.0818	0.811	-0.1818	0.5926
	Frontal DWML	0.1182	0.7293	-0.0364	0.9153
	Parieto-occipital DWML	-0.0818	0.811	-0.1909	0.5739
Middle frontal	Anterior horn PVWML	0.1	0.7699	0.0091	0.9788
	Posterior horn PVWML	0.1545	0.65	0.2182	0.5192
	Frontal DWML	0.0182	0.9577	0.2232	0.5094
	Parieto-occipital DWML	0.0455	0.8944	0.0455	0.8944
Medial frontal	Anterior horn PVWML	-0.0455	0.8944	-0.1091	0.7495
	Posterior horn PVWML	-0.0818	0.811	-0.0727	0.8317
	Frontal DWML	0.1091	0.7495	0.0592	0.8627
	Parieto-occipital DWML	-0.1091	0.7495	-0.1091	0.7495
Precuneus	Anterior horn PVWML	-0.2636	0.4334	-0.2000	0.5554
	Posterior horn PVWML	-0.2091	0.5372	-0.0727	0.8317
	Frontal DWML	-0.1909	0.5739	-0.1412	0.6787
	Parieto-occipital DWML	-0.2909	0.3855	-0.2909	0.3855
Medial temporal	Anterior horn PVWML	0.2	0.5554	0.2182	0.5192
	Posterior horn PVWML	0.2455	0.4669	0.3909	0.2345
	Frontal DWML	0.1545	0.65	0.369	0.2641
	Parieto-occipital DWML	0.2182	0.5192	0.2818	0.4011
PCC	Anterior horn PVWML	0.0727	0.8317	0.2364	0.4841
	Posterior horn PVWML	0.2545	0.45	0.3909	0.2345

Frontal DWML	-0.1455	0.6696	0.3052	0.3614
Parieto-occipital DWML	-0.0727	0.8317	0.1909	0.5739

rhGM: regional hypoperfusion of gray matter; PVWML: periventricular white matter lesion; DWML: deep white matter lesion; PCC: posterior cingulate cortex.

Supplemental Table 6. Statistical data of Spearman's multivariate analysis for the connectivity between rhGM and WML in each symptom of subcategories of BPSD in AD type dementia and aMCI.

		AD type				aMCI			
		Left		Right		Left		Right	
rhGM	WML	rs	p	rs	p	rs	p	rs	p
**Hypoactivity**									
Superior frontal	Anterior horn PVWML	0.2585	0.1027	0.208	0.1919	0.4406	0.1517	0.2517	0.4299
	Posterior horn PVWML	0.1087	0.4987	-0.0370	0.8182	0.4266	0.1667	0.2238	0.4845
	Frontal DWML	0.3305	0.0348*	0.2295	0.149	0.4238	0.1698	0.4507	0.1414
	Parieto-occipital DWML	0.2232	0.1607	0.0864	0.5911	0.5105	0.0899	0.1748	0.5868
Middle frontal	Anterior horn PVWML	0.2559	0.1063	0.166	0.2995	0.4196	0.1745	0.4406	0.1517
	Posterior horn PVWML	0.0172	0.9148	-0.0649	0.6869	0.4406	0.1517	0.3147	0.3191
	Frontal DWML	0.3066	0.0512	0.2159	0.1753	0.5114	0.0893	0.6338	0.0269*
	Parieto-occipital DWML	0.1216	0.4488	0.0148	0.9268	0.4406	0.1517	0.3287	0.2969
Medial frontal	Anterior horn PVWML	0.2218	0.1634	0.1897	0.2348	0.4755	0.1182	0.4196	0.1745
	Posterior horn PVWML	0.0779	0.6284	-0.0225	0.8891	0.3566	0.2551	0.2937	0.3541
	Frontal DWML	0.2947	0.0614	0.2359	0.1376	0.4729	0.1205	0.5775	0.0493*
	Parieto-occipital DWML	0.2186	0.1696	0.0704	0.6619	0.3916	0.2081	0.2517	0.4299

Precuneus	Anterior horn PVWML	0.1253	0.4352	0.1479	0.3561	-0.2378	0.4568	-0.2028	0.5273
	Posterior horn PVWML	-0.0134	0.9337	-0.0207	0.8976	-0.0909	0.7787	-0.0979	0.7621
	Frontal DWML	0.2556	0.1067	0.0734	0.6483	-0.2067	0.5193	-0.2465	0.4399
	Parieto-occipital DWML	0.1772	0.2678	0.092	0.5673	-0.0629	0.8459	-0.3497	0.2652
Medial temporal	Anterior horn PVWML	0.2055	0.1974	0.1425	0.3741	0.4755	0.1182	0.2448	0.4433
	Posterior horn PVWML	0.1327	0.4083	-0.0413	0.7977	0.2937	0.3541	-0.0839	0.7954
	Frontal DWML	0.2448	0.1229	0.1451	0.3655	0.4799	0.1144	0.4437	0.1485
	Parieto-occipital DWML	0.2202	0.1665	0.0448	0.781	0.2727	0.3911	0.028	0.9312
PCC	Anterior horn PVWML	0.0984	0.5404	0.0563	0.7268	0.3566	0.2551	0.4266	0.1667
	Posterior horn PVWML	0.073	0.6501	0.0816	0.6119	0.2727	0.3911	0.2517	0.4299
	Frontal DWML	0.1009	0.5303	0.0389	0.8093	0.3783	0.2253	0.5141	0.0873
	Parieto-occipital DWML	0.0679	0.673	0.1436	0.3706	0.1399	0.6646	0.1958	0.5419
**Hyperactivity**									
Superior frontal	Anterior horn PVWML	-0.3699	0.1084	-0.4030	0.0781	-0.4667	0.2054	-0.5333	0.1392
	Posterior horn PVWML	-0.3008	0.1976	-0.2782	0.235	-0.3833	0.3085	-0.7500	0.0199*
	Frontal DWML	-0.1158	0.6269	-0.2505	0.2868	0.05	0.8984	-0.3431	0.366
	Parieto-occipital DWML	-0.3263	0.1603	-0.2737	0.243	-0.3167	0.4064	-0.7833	0.0125*

Middle frontal	Anterior horn PVWML	-0.4556	0.0435*	-0.5053	0.0231*	-0.6167	0.0769	-0.5167	0.1544
	Posterior horn PVWML	-0.4677	0.0376*	-0.4000	0.0806	-0.6000	0.0876	-0.7500	0.0199*
	Frontal DWML	-0.2286	0.3324	-0.4152	0.0687	-0.1833	0.6368	-0.4519	0.222
	Parieto-occipital DWML	-0.4586	0.0420*	-0.3323	0.1523	-0.5333	0.1392	-0.7833	0.0125*
Medial frontal	Anterior horn PVWML	-0.4722	0.0355*	-0.4917	0.0277*	-0.5500	0.125	-0.5833	0.0992
	Posterior horn PVWML	-0.4271	0.0604	-0.3549	0.1247	-0.5667	0.1116	-0.8500	0.0037*
	Frontal DWML	-0.2902	0.2145	-0.3956	0.0842	-0.0833	0.8312	-0.4100	0.273
	Parieto-occipital DWML	-0.4376	0.0537	-0.3759	0.1023	-0.4667	0.2054	-0.8667	0.0025*
Precuneus	Anterior horn PVWML	-0.4451	0.0492*	-0.2767	0.2376	-0.0500	0.8984	-0.1667	0.6682
	Posterior horn PVWML	-0.4075	0.0745	-0.2331	0.3227	0.1833	0.6368	0	1
	Frontal DWML	-0.1744	0.462	-0.3791	0.0993	0.2	0.6059	-0.2008	0.6044
	Parieto-occipital DWML	-0.4316	0.0574	-0.2211	0.349	-0.0500	0.8984	-0.1667	0.6682
Medial temporal	Anterior horn PVWML	-0.1880	0.4274	-0.2992	0.1999	-0.4000	0.2861	-0.1833	0.6368
	Posterior horn PVWML	-0.2346	0.3195	-0.1714	0.4699	-0.4833	0.1875	-0.4833	0.1875
	Frontal DWML	0.0105	0.9649	-0.1143	0.6313	-0.1667	0.6682	-0.1004	0.7971
	Parieto-occipital DWML	-0.1188	0.6179	-0.1549	0.5144	-0.4833	0.1875	-0.4833	0.1875
PCC	Anterior horn PVWML	-0.3368	0.1464	-0.2917	0.212	-0.2167	0.5755	0.0667	0.8647

	Posterior horn PVWML	-0.3534	0.1264	-0.1895	0.4237	-0.1000	0.798	-0.1667	0.6682
	Frontal DWML	-0.0917	0.7005	-0.1557	0.5122	0.1667	0.6682	0.3096	0.4175
	Parieto-occipital DWML	-0.3038	0.1929	-0.1203	0.6134	-0.0667	0.8647	-0.1667	0.6682
**Hallucination/delusion**									
Superior frontal	Anterior horn PVWML	0.1789	0.4503	0.018	0.9398				
	Posterior horn PVWML	0.1278	0.5912	-0.0647	0.7865				
	Frontal DWML	0.2287	0.3322	0.0075	0.9749				
	Parieto-occipital DWML	0.218	0.3557	-0.0421	0.8601				
Middle frontal	Anterior horn PVWML	0.0707	0.7672	0.0556	0.8158				
	Posterior horn PVWML	-0.0090	0.9699	0.0782	0.7431				
	Frontal DWML	0.0755	0.7518	0.0648	0.7862				
	Parieto-occipital DWML	-0.0165	0.9448	0.003	0.99				
Medial frontal	Anterior horn PVWML	0.0707	0.7672	-0.0647	0.7865				
	Posterior horn PVWML	-0.0632	0.7914	-0.0256	0.9148				
	Frontal DWML	0.114	0.6324	-0.0602	0.8008				
	Parieto-occipital DWML	0.1248	0.6001	-0.0331	0.8899				
Precuneus	Anterior horn PVWML	-0.0812	0.7336	-0.1504	0.5269				

	Posterior horn PVWML	-0.1173	0.6224	0.0331	0.8899
	Frontal DWML	0.0513	0.8299	-0.1084	0.6491
	Parieto-occipital DWML	0.0256	0.9148	-0.0135	0.9548
Medial temporal	Anterior horn PVWML	0.1444	0.5437	0.1023	0.6678
	Posterior horn PVWML	0.3729	0.1053	0.2678	0.2537
	Frontal DWML	0.3525	0.1275	0.3104	0.1829
	Parieto-occipital DWML	0.4466	0.0484*	0.2279	0.3338
PCC	Anterior horn PVWML	-0.2180	0.3557	-0.1594	0.5021
	Posterior horn PVWML	-0.0827	0.7289	0.015	0.9498
	Frontal DWML	-0.0717	0.7639	-0.0512	0.8302
	Parieto-occipital DWML	-0.0466	0.8453	0.0165	0.9448

rhGM: regional hypoperfusion of gray matter; PVWML: periventricular white matter lesion; DWML: deep white matter lesion; PCC: posterior cingulate cortex.
